# Modified blood cell GAP model as a prognostic biomarker in idiopathic pulmonary fibrosis

**DOI:** 10.1183/23120541.00666-2023

**Published:** 2024-07-29

**Authors:** Michael Kreuter, Joyce S. Lee, Argyrios Tzouvelekis, Justin M. Oldham, Philip L. Molyneaux, Derek Weycker, Mark Atwood, Katerina Samara, Klaus-Uwe Kirchgässler, Toby M. Maher

**Affiliations:** 1 Center for Pulmonary Medicine, Departments of Pneumology, Mainz University Medical Center, and of Pulmonary, Critical Care and Sleep Medicine, Marienhaus Clinic Mainz, Mainz, Germany; 2 Department of Medicine, University of Colorado, Denver, CO, USA; 3 Department of Respiratory Medicine, University of Patras, Patras, Greece; 4 Division of Pulmonary and Critical Care Medicine, University of Michigan, Ann Arbor, MI, USA; 5 Interstitial Lung Disease Unit, Royal Brompton and Harefield Hospitals, Guy's and St Thomas’ NHS Foundation Trust, London, UK; 6 National Heart and Lung Institute, Imperial College London, London, UK; 7 Avalere Health, Boston, MA, USA; 8 F. Hoffmann-La Roche, Ltd., Basel, Switzerland; 9 Hastings Center for Pulmonary Research and Division of Pulmonary, Critical Care, and Sleep Medicine, Keck School of Medicine, University of Southern California, Los Angeles, CA, USA

## Abstract

**Background:**

The Gender, Age and Physiology (GAP) model is a simple mortality prediction tool in patients with idiopathic pulmonary fibrosis that uses demographic and physiological variables available at initial evaluation. White blood cell variables may have associations with idiopathic pulmonary fibrosis outcomes. We evaluated whether incorporating blood cell counts in modified GAP (cGAP) models would improve outcome prediction in patients with idiopathic pulmonary fibrosis.

**Patients and methods:**

This retrospective analysis included pooled data from phase 3 randomised trials of pirfenidone in idiopathic pulmonary fibrosis (ASCEND, CAPACITY 004, CAPACITY 006). Study outcomes (disease progression, all-cause mortality, all-cause hospitalisation, respiratory-related hospitalisation) were evaluated during the initial 1-year period. Shared frailty models were used to evaluate associations between continuous and categorical baseline white and red blood cell parameters and study outcomes in a bivariate context, and to evaluate the impact of adding continuous monocyte count (cGAP1) or white and red blood cell parameters (cGAP2) to traditional GAP variables in a multivariable context based on C-statistics changes.

**Results:**

Data were pooled from 1247 patients (pirfenidone, n=623; placebo, n=624). Significant associations (bivariate analyses) were idiopathic pulmonary fibrosis progression with neutrophil and eosinophil counts; all-cause mortality with monocyte and neutrophil counts; all-cause hospitalisation with monocyte count, neutrophil count and haemoglobin level; and respiratory-related hospitalisation with monocyte count, neutrophil count and haemoglobin level. In multivariate analyses, C-statistics were highest for the cGAP2 model for each of the outcomes.

**Conclusion:**

Modified GAP models incorporating monocyte counts alone or plus other white and red blood cell variables may be useful to improve prediction of outcomes in patients with idiopathic pulmonary fibrosis.

## Shareable abstract

**The Gender-Age-Physiology (GAP) index can be modified to incorporate monocyte counts alone or plus other white blood cell and red blood cell variables, and has the potential for improving predictability of outcomes in patients with idiopathic pulmonary fibrosis** https://bit.ly/3wCvRjb

## Introduction

Idiopathic pulmonary fibrosis (IPF) is a chronic, fibrosing interstitial lung disease that is characterised by progressive worsening of lung function, loss of quality of life and dyspnoea, and has a poor prognosis [1, 2]. Two antifibrotics, pirfenidone and nintedanib, are approved for the treatment of IPF; they are associated with reductions in lung function decline and can potentially improve survival [3–8].

The disease course of IPF is highly variable, making individual patient prognosis difficult to predict [9]. Consequently, there is a need for simple prognostic tools that can help clinicians to identify patients at risk of rapid IPF progression and poor outcomes. The traditional Gender, Age and Physiology (GAP) model, which was developed as an easy-to-use tool for predicting the risk of mortality in IPF, uses four variables that are usually available at the time of the initial evaluation: gender, age and physiology, as measured by forced vital capacity (FVC) and carbon monoxide diffusing capacity of the lung (*D*_L__CO_) [10]. The GAP model has been validated in various patient populations with IPF [10–13]. However, the traditional version of the model has several potential limitations, including the exclusion of additional baseline variables that were not considered at the time of model development, the lack of consideration of longitudinal disease behaviour and the fact that it may overestimate risk, especially in lower risk groups [10, 11]. Additionally, the GAP model is not used to directly predict outcomes other than mortality, such as IPF progression and hospitalisation, and shows worse performance for predicting such outcomes (*e.g.* decline in % predicted FVC) compared with mortality [14].

Several studies have shown that modifying the traditional GAP model to incorporate a wider range of variables (*e.g.* exercise parameters, radiology results, biomarkers or comorbidities) may provide additional prognostic value in IPF [15–18]. Previous studies have also shown that certain white blood cell (WBC) parameters, particularly monocyte counts, may be associated with IPF outcomes [19–25]. For example, a retrospective, pooled analysis of data from four phase 3 trials in patients with IPF found that elevated monocyte counts were significantly associated with increased risks of IPF progression, all-cause mortality and all-cause hospitalisation [20]. Therefore, we hypothesised that inclusion of blood cell counts in a modified GAP model may improve its predictive performance. In this study, we compared the predictive value of the traditional GAP model with modified GAP models that incorporate WBC and/or red blood cell (RBC) parameters (cGAP models) for various IPF outcomes.

## Materials and methods

### Study design

This retrospective, *post hoc* analysis included pooled data from three phase 3, randomised, placebo-controlled, double-blind trials of pirfenidone in patients with IPF: ASCEND (ClinicalTrials.gov: NCT01366209) and the two CAPACITY studies (study 004: NCT00287716 and study 006: NCT00287729) [3, 4]. The trials were conducted in accordance with the Declaration of Helsinki and International Conference on Harmonization Good Clinical Practice Guidelines. All patients provided written informed consent before participation. Study protocols were approved by the institutional review board/ethics committee at each centre.

### Study subjects

Full eligibility criteria for the ASCEND and CAPACITY trials have been described previously [3, 4]. Both trials recruited patients aged 40–80 years with a diagnosis of IPF confirmed by high-resolution computed tomography (HRCT) alone or HRCT plus surgical lung biopsy. Eligible patients had a % predicted FVC ≥50% (and ≤90% in ASCEND), % predicted *D*_LCO_ ≥35% (≥30% and ≤90% in ASCEND) and a 6-min walk distance (6MWD) ≥150 m, with no evidence of improvement in IPF disease severity over the preceding year. Patients who received 2403 mg·day^−1^ pirfenidone or placebo were included in this *post hoc* analysis, whereas patients who received 1197 mg·day^−1^ pirfenidone in CAPACITY 004 were excluded in line with several previous analyses that have been performed without including these patients [24, 26–28].

### Methods

The population characteristics evaluated using data collected at the trial baseline visit included GAP parameters (gender, age, FVC, *D*_LCO_), WBC counts (monocytes, lymphocytes, neutrophils, basophils, eosinophils), RBC parameters (mean corpuscular haemoglobin (Hb) in pg, mean corpuscular Hb concentration in g·L^−1^, erythrocytes, Hb, platelets) and selected other variables (6MWD, dyspnoea measured using the University of California–San Diego Shortness of Breath Questionnaire, comorbidity profile, cardiovascular risk factors and immunosuppressant use (evidence of concomitant immunosuppressant use with Anatomical Therapeutic Chemical classification level 2/3)). WBC and RBC parameters were defined as continuous variables and, alternatively, as categorical variables (below normal, normal or above normal, based on published literature (monocyte counts) or published ranges (other WBC and RBC parameters)) [20, 29–32].

Study outcomes were evaluated during the 1-year period following trial baseline visits and included IPF progression, defined as a ≥10% decline in % predicted FVC, a ≥50 m decline in 6MWD or death (declines in FVC and 6MWD required confirmation at two consecutive assessments ≥6 weeks apart); all-cause mortality; all-cause hospitalisation; and respiratory-related hospitalisation, where the primary reason for admission was respiratory related as determined by investigators.

### Analysis

Characteristics of the study population at the trial baseline visit were summarised descriptively. The association between baseline WBC and RBC parameters (continuous definitions) and study outcomes were evaluated in a bivariate context using shared frailty models (an extension of the Cox proportional hazards model that adjusts for intra-cluster (intra-trial) correlation). The number of events per variable are detailed for each outcome as recommended for the conduct and reporting of proportional hazard analysis [33, 34]. Shared frailty models were also employed in a multivariate context to examine the impact of adding continuous monocyte counts (cGAP1) or continuous WBC and RBC parameters (cGAP2) to variables included in the traditional GAP index (GAP). For each multivariable model, C-statistics were estimated, along with the change in C-statistics for GAP *versus* cGAP1 and cGAP1 *versus* cGAP2; 95% confidence intervals were calculated *via* non-parametric bootstrapping (1000 replications). Although it is possible to use categorical variables for C-statistical analysis to assess nonlinear relationships, continuous variables were used in this study in line with previous analyses in GAP parameters.

Kaplan–Meier curves were constructed for predicted survival probabilities over time for IPF progression and all-cause mortality for the GAP, cGAP1 and cGAP2 models. For each patient in the study population, survival probabilities for IPF progression and all-cause mortality, respectively, were predicted *via* each of the three models (GAP/cGAP1/cGAP2) and predicted probabilities were subsequently grouped into tertiles. Tertiles were then used as strata for the respective Kaplan–Meier curves. Patients were censored at the time of loss to follow-up, lung transplantation or end of the 1-year follow-up period, whichever occurred first.

Additional analyses were conducted considering categorical definitions for WBC and RBC parameters, treatment (pirfenidone *versus* placebo) and other patient characteristics (6MWD, dyspnoea, current/former smoker, medical history).

## Results

### Patients

Data were pooled from 1247 patients with IPF who received 2403 mg·day^−1^ pirfenidone (n=623) or placebo (n=624) in the ASCEND or CAPACITY phase 3 trials (table 1). Overall, 74.4% of patients were male, mean age was 67.2 years, mean±sd % predicted FVC was 71.8±13.4% and mean±sd % predicted *D*_LCO_ was 45.6±10.7%. Baseline WBC and RBC parameters are summarised in table 1. Immunosuppressants were used by 62.7% of patients (includes use for any duration, dose or indication), including steroids (62.6%) and/or non-steroids (4.8%).

**TABLE 1:** Baseline characteristics of the study population

	Overall
**Participants (n)**	1247
**Male sex**	928 (74.4)
**Age (years)**	67.2±7.5
**FVC (% predicted)**	71.8 (13.4)
***D*_LCO_ (% predicted)**	45.6±10.7
Missing	2 (0.2)
**6MWD (m)**	407.8±94.0
Missing	15 (1.2)
**UCSD-SOBQ**	34.6±21.5
Missing	13 (1.0)
**WBC counts**	
Monocytes (×10^9^·L^−1^)	0.49±0.17
Lymphocytes (×10^9^·L^−1^)	2.11±0.71
Neutrophils (×10^9^·L^−1^)	5.19±1.72
Basophils (×10^9^·L^−1^)	0.06±0.03
Eosinophils (×10^9^·L^−1^)	0.25±0.16
**RBC parameters**	
Mean corpuscular Hb (pg)	30.59±2.02
Mean corpuscular Hb (g·L^−1^)	334.72±13.01
Erythrocytes (×10^12^·L^−1^)	4.63±0.71
Hb (g·L^−1^)	141.57±13.39
Platelets (g·L^−1^)	246.71±63.86
**Comorbidities**	
Gastroesophageal reflux disease	641 (51.4)
Coronary artery disease	280 (22.5)
Myocardial infarction	71 (5.7)
Chronic obstructive pulmonary disease	44 (3.5)
Pulmonary hypertension	33 (2.6)
Chronic renal failure	32 (2.6)
Deep vein thrombosis	30 (2.4)
Arteriosclerosis	16 (1.3)
Pulmonary embolism	15 (1.2)
Congestive heart failure	14 (1.1)
**Cardiovascular risk factors**	
Smoker (current/former)	790 (63.4)
Hypertension	651 (52.2)
Hypercholesterolaemia	605 (48.5)
Obesity, BMI >30 kg·m^−2^	549 (44.0)
Diabetes	285 (22.9)
**Immunosuppressant use** ^#^	
Steroid	780 (62.6)
Non-steroid	60 (4.8)
Neither	465 (37.3)

Data are presented as mean±sd or n (%), unless otherwise indicated. FVC: forced vital capacity; *D*_Lco_: diffusing capacity of the lung for carbon monoxide; 6MWD: 6-min walk distance; UCSD-SOBQ: University of California–San Diego Shortness of Breath Questionnaire; WBC: white blood cell; RBC: red blood cell; Hb: haemoglobin; BMI: body mass index. ^#^: use of concomitant medications was limited by both the ASCEND and CAPACITY protocols and does not necessarily reflect chronic use. Includes use for non-idiopathic pulmonary fibrosis (IPF) indications (ASCEND) or protocol-defined IPF acute exacerbations, acute respiratory decompensation or progression of disease (CAPACITY). Data represent the number of patients reporting any immunosuppressant use during the studies, independent of duration, dose and indication.

### Study outcomes by baseline WBC and RBC parameters: bivariate analyses

Table 2 presents bivariate analyses for the study outcomes over 1 year using continuous baseline WBC and RBC variables. IPF progression was significantly associated with baseline neutrophil count (hazard ratio (HR) 1.09, p<0.001) and eosinophil count (HR 2.19, p=0.003). All-cause mortality was significantly associated with baseline monocyte count (HR 8.43, p<0.001) and neutrophil count (HR 1.22, p<0.001). All-cause hospitalisation and respiratory-related hospitalisation were each significantly associated with baseline monocyte count (HR 3.23 and HR 3.72, respectively; p<0.001 and p=0.001), neutrophil count (HR 1.13 and HR 1.15, respectively; both p<0.001) and Hb level (both HR 0.99; p=0.004 and p=0.013, respectively).

**TABLE 2:** Bivariate analyses of white blood cell/red blood cell parameters (continuous)

	Mean±sd	Hazard ratios (95% CI) by study outcome^#^			
		IPF progression^¶^	All-cause mortality^+^	All-cause hospitalisation^§^	Respiratory hospitalisation^ƒ^
**Monocytes (×10^9^·L^−1^)**	0.5±0.2	1.31 (0.76–2.27)	8.43 (2.91–24.46)***	3.23 (1.64–6.37)***	3.72 (1.66–8.36)**
**Lymphocytes (×10^9^·L^−1^)**	2.1±0.7	0.95 (0.83–1.08)	0.83 (0.58–1.20)	1.06 (0.88–1.27)	1.07 (0.86–1.32)
**Neutrophils (×10^9^·L^−1^)**	5.2±1.7	1.09 (1.04–1.15)***	1.22 (1.11–1.34)***	1.13 (1.06–1.21)***	1.15 (1.06–1.24)***
**Basophils (×10^9^·L^−1^)**	0.1±0.0	11.12 (0.65–190.89)	0.37 (0.00–>999)	4.86 (0.08–296.70)	4.40 (0.03–696.68)
**Eosinophils (×10^9^·L^−1^)**	0.3±0.2	2.19 (1.32–3.65)**	3.21 (0.99–10.41)	1.53 (0.72–3.23)	1.71 (0.70–4.16)
**Corpuscular Hb (pg)**	30.6±2.0	1.00 (0.96–1.05)	1.04 (0.93–1.18)	0.94 (0.88–1.00)	0.93 (0.86–1.00)
**Corpuscular Hb (g·L^−1^)**	334.7±13.0	1.00 (0.99–1.00)	1.00 (0.98–1.02)	0.99 (0.98–1.00)	0.99 (0.98–1.01)
**Erythrocytes (×10^12^·L^−1^)**	4.6±0.7	0.90 (0.78–1.04)	0.84 (0.59–1.21)	0.92 (0.75–1.12)	0.94 (0.76–1.17)
**Hb (g·L^−1^)**	141.6±13.4	1.00 (0.99–1.00)	0.99 (0.97–1.01)	0.99 (0.98–1.00)**	0.99 (0.97–1.00)*
**Platelets (×10^9^·L^−1^)**	246.7±63.9	1.00 (1.00–1.00)	1.00 (1.00–1.01)	1.00 (1.00–1.00)	1.00 (1.00–1.00)

IPF: idiopathic pulmonary fibrosis; Hb: haemoglobin. **^#^**: n=1247 for all variables; follow-up between visits was 12–13 weeks; **^¶^**: 429 events (34.4% of patients); **^+^**: 64 events (5.1% of patients); ^§^: 219 events (17.6% of patients); **^ƒ^**: 155 events (12.4% of patients). *: p<0.05; **: p<0.01; ***: p<0.001.

In the bivariate analyses for study outcomes using categorical baseline WBC and RBC definitions (below normal, normal, above normal), the WBC and RBC categories showing statistical significance were similar to those identified in the continuous analyses (supplementary table S1), although some additional associations were identified in the categorical analysis (all-cause mortality: mean corpuscular Hb concentration in g·L^−1^; all-cause hospitalisation: mean corpuscular Hb in pg and erythrocyte count; respiratory-related hospitalisation: mean corpuscular Hb in pg).

### Study outcomes by GAP and cGAP models: multivariable analyses

The prognostic value of each GAP and cGAP model for study outcomes over 1 year was evaluated using C-statistics generated from the multivariable shared frailty models (table 3). Hazard ratios from the shared frailty models for individual parameters within each GAP and cGAP (cGAP1 and cGAP2) model are shown in supplementary table S2 (continuous variables) and supplementary table S3 (categorical variables). All individual variables contributed to estimation of the C-statistics, regardless of whether they were statistically significant or not.

**TABLE 3:** Change in C-statistic with the addition of white blood cell/red blood cell parameters (continuous) to the GAP model

Model specification	IPF progression	All-cause mortality	All-cause hospitalisation	Respiratory hospitalisation
**GAP**				
C-statistic (95% CI)	0.595 (0.588–0.596)	0.730 (0.705–0.725)	0.624 (0.615–0.621)	0.603 (0.599–0.608)
**cGAP1**				
C-statistic (95% CI)	0.595 (0.588–0.596)	0.740 (0.721–0.734)	0.630 (0.623–0.628)	0.607 (0.602–0.611)
Change *versus* GAP (95% CI)	0.000 (–0.001–0.000)	0.010 (0.009–0.016)	0.006 (0.007–0.009)	0.004 (0.002–0.005)
**cGAP2**				
C-statistic (95% CI)	0.621 (0.613–0.615)	0.765 (0.730–0.755)	0.645 (0.629–0.640)	0.635 (0.620–0.638)
Change *versus* cGAP1 (95% CI)	0.026 (0.019–0.026)	0.025 (0.009–0.026)	0.016 (0.005–0.012)	0.028 (0.018–0.027)

IPF: idiopathic pulmonary fibrosis; GAP: Gender, Age and Physiology; cGAP1: GAP model with addition of continuous monocyte counts; cGAP2: GAP model with addition of continuous monocyte counts and other white and red blood cell parameters, including lymphocytes, neutrophils, basophils, eosinophils, mean corpuscular haemoglobin (Hb), mean corpuscular Hb concentration, erythrocytes, Hb and platelets.

C-statistics were highest for the cGAP2 model for each of the outcomes. The respective C-statistics for the traditional GAP model, cGAP1 and cGAP2 were 0.595, 0.595 and 0.621 for IPF progression; 0.730, 0.740 and 0.765 for all-cause mortality; 0.624, 0.630 and 0.645 for all-cause hospitalisation; and 0.603, 0.607 and 0.635 for respiratory-related hospitalisation. Changes in C-statistics for GAP *versus* cGAP1 and cGAP1 *versus* cGAP2 are shown in table 3.

**FIGURE 1:**
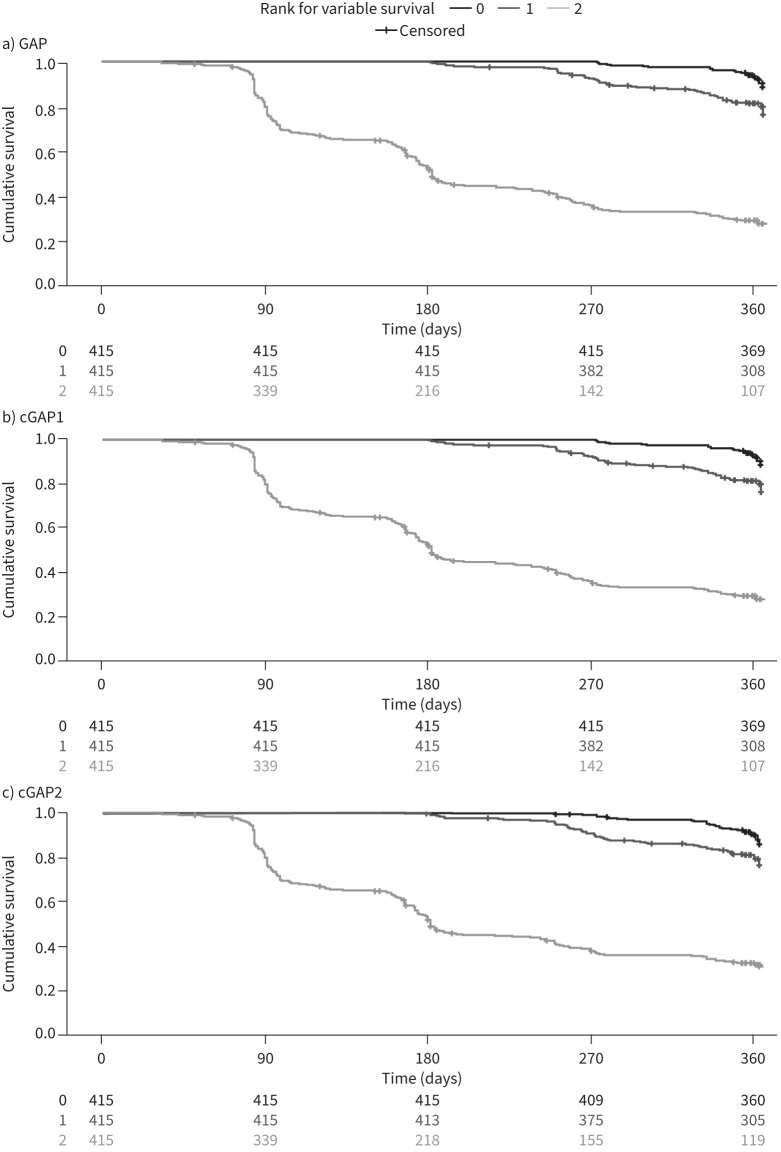
Kaplan–Meier curves for idiopathic pulmonary fibrosis progression, by tertile scores for the a) Gender, Age, Physiology (GAP), b) modified Gender, Age, Physiology 1 (cGAP1) and c) modified Gender, Age, Physiology 2 (cGAP2) models.

Kaplan–Meier curves for IPF progression and all-cause mortality by tertile scores for each of the models are shown in figure 1 and supplementary figure S1, respectively. For IPF progression, clear separation of the third tertile from the first and second tertiles was seen from the start for all three models, with separation between the first and second tertiles observed after the first 6 months. For all-cause mortality, poorer overall separation between the tertiles was observed, again with a similar pattern across all three models.

Multivariable analyses of WBC and RBC parameters and study outcomes (where independent variables were determined using a backwards selection model), including pirfenidone *versus* placebo as variables, are summarised in table 4. Results for the fully specified model are presented in supplementary tables S4 and S5.

**TABLE 4:** Multivariable analyses of WBC/RBC parameters (continuous) and other parameters (WBC/RBC results only)

Independent variables	Hazard ratios (95% CI) by study outcome			
	IPF progression^#^	All-cause mortality^¶^	All-cause hospitalisation^+^	Respiratory hospitalisation^§^
**Monocytes (×10^9^·L^−1^)**	0.77 (0.40–1.50)	4.14 (0.85–20.19)	1.62 (0.70–3.79)	1.53 (0.56–4.18)
**Lymphocytes (×10^9^·L^−1^)**	0.91 (0.79–1.06)	0.83 (0.56–1.24)	1.01 (0.83–1.23)	1.10 (0.88–1.39)
**Neutrophils (×10^9^·L^−1^)**	1.06 (1.00–1.13)*	1.06 (0.92–1.22)	1.05 (0.97–1.15)	1.07 (0.97–1.18)
**Basophils (×10^9^·L^−1^)**	5.93 (0.22–158.05)	0.94 (0.00–>999)	2.80 (0.02–327.96)	2.98 (0.01–922.07)
**Eosinophils (×10^9^·L^−1^)**	2.14 (1.20–3.80)*	2.08 (0.52–8.30)	0.94 (0.39–2.28)	0.81 (0.27–2.47)
**Mean corpuscular Hb (pg)**	1.04 (0.97–1.10)	1.01 (0.86–1.19)	0.93 (0.85–1.01)	0.89 (0.80–0.99)
**Mean corpuscular Hb (g·L^−1^)**	1.00 (0.99–1.00)	1.01 (0.99–1.03)	1.00 (0.99–1.01)	0.99 (0.98–1.01)
**Erythrocytes (×10^12^·L^−1^)**	1.01 (0.84–1.22)	1.01 (0.61–1.69)	0.93 (0.69–1.26)	0.89 (0.59–1.34)
**Hb (g·L^−1^)**	0.99 (0.98–1.00)	0.98 (0.95–1.01)	0.99 (0.98–1.01)	0.99 (0.97–1.01)
**Platelets (×10^9^·L^−1^)**	1.00 (1.00–1.00)	1.00 (1.00–1.01)	1.00 (1.00–1.00)	1.00 (1.00–1.00)

n=1247 for all variables; all blood cell variables were forced into the model. Follow-up between visits was 12–13 weeks. Other model specifications were determined *via* backward selection from the following candidate variables: treatment (pirfenidone *versus* placebo), age, male, current/former smoker, forced vital capacity (% predicted, time-dependent), diffusing capacity of the lung for carbon monoxide (time-dependent), 6-min walk distance (time-dependent), University of California–San Diego Shortness of Breath Questionnaire (time-dependent), pulmonary hypertension, pulmonary embolism, gastro-oesophageal reflux disease, deep vein thrombosis, chronic obstructive pulmonary disease, arteriosclerosis, chronic renal failure, coronary artery disease, myocardial infarction, chronic heart failure, hypertension, hypercholesterolaemia, obesity, infections and steroid use. WBC: white blood cell; RBC: red blood cell; IPF: idiopathic pulmonary fibrosis; Hb: haemoglobin. **^#^**: 429 events (34.4% of patients); **^¶^**: 64 events (5.1% of all patients); ^+^: 219 events (17.6% of all patients); ^§^: 155 events (12.4% of all patients). *: p<0.05; **: p<0.01; ***: p<0.001.

## Discussion

To our knowledge, our study is the first to investigate such a broad range of WBC and RBC variables as prognostic biomarkers in patients with IPF. In bivariate analyses, significant associations between baseline WBC and RBC parameters and 1-year outcomes were found for IPF progression and neutrophil and eosinophil counts; all-cause mortality and monocyte and neutrophil counts; all-cause hospitalisations and monocyte counts, neutrophil counts and Hb; and respiratory-related hospitalisations and monocyte counts, neutrophil counts and Hb. Comorbidities significantly influence the clinical course of IPF but their prognostic value is not fully understood [35]. Comorbidities leading to elevated monocyte count may also be linked to increased mortality and hospitalisations (including those related to acute IPF exacerbations). While these factors may not have directly triggered FVC or 6MWD decline, monocyte count may be associated with IPF survival through one or more non-causal pathways and could represent an epiphenomenon. Overall, our findings are largely in line with previous studies describing specific WBC-related biomarkers in IPF. In one study, patients with higher total WBC counts had significantly shorter transplant-free survival than those with lower total WBC counts [19]. However, in contrast to the current study, counts for individual WBC types were not evaluated. Several studies have also reported significant associations between elevated monocyte counts and all-cause mortality [20–22], which is consistent with our findings. Kreuter *et al.* [20] investigated additional IPF outcomes and found significant associations between elevated monocyte counts and IPF progression and all-cause hospitalisation. Likewise, Teoh *et al.* [22] found that elevated neutrophil counts were significantly associated with all-cause mortality, which is in agreement with the current study. Significant associations have also been identified between the presence of IPF and the neutrophil/lymphocyte ratio, derived neutrophil/lymphocyte ratio and monocyte/lymphocyte ratio [23]. Moreover, the results of another study suggested that patients with the greatest change in neutrophil/lymphocyte ratio or platelet/lymphocyte ratio over 12 months may be at an increased risk of IPF progression, all-cause mortality and respiratory hospitalisation [24]. In contrast to WBC parameters, there is a paucity of data regarding potential RBC-related prognostic biomarkers in IPF. One study reported an association of high red cell distribution width with more advanced disease at baseline (lower median % predicted FVC and increased need for long-term oxygen therapy). However, red cell distribution width did not predict disease progression or all-cause mortality [25]. It is notable, therefore, that we identified significant associations between hospitalisations (particularly respiratory-related hospitalisations) and Hb parameters.

In patients with IPF, modifications of the traditional GAP model to include monocyte counts (cGAP1) or monocyte counts plus other WBC and RBC variables (cGAP2) modestly improved predictions of study outcomes. For all four clinical outcomes (IPF progression, all-cause mortality, and all-cause and respiratory-related hospitalisations), the cGAP2 model had the highest nominal C-statistics, demonstrating improved C-statistics over the GAP and cGAP1 models. Across all three models, the highest C-statistic values were produced for all-cause mortality, followed by all-cause hospitalisation. However, Kaplan–Meier curves of IPF progression by tertile showed greater separation than those for all-cause mortality. Potentially, this reflects the high percentage of patients (>35%) experiencing IPF progression during follow-up, which may make it challenging to differentiate patients at low *versus* high risk of progression. Moreover, it is difficult to draw conclusions on mortality outcomes after only 1 year of follow-up and with a small number of patients. Survival curves for both IPF progression and all-cause mortality were largely similar across the three GAP models. While these results demonstrate that adding variables such as monocytes to the original GAP model can improve predictions, it does not solve the fundamental issues with the model: the GAP model was originally designed to predict mortality and not other outcomes such as acute deterioration or physiological progression [10].

Our results demonstrate proof of concept that a multidimensional prognostic approach may aid in clinical decision-making. In that regard, cGAP2 may be useful for both evaluating patients with new diagnosis of IPF and identifying patients with fast progression or higher risk of mortality who would be prime candidates for early intervention or transplant evaluation. In addition to potentially improving the predictive value of the cGAP model, incorporating a broad range of WBC and RBC parameters in the cGAP2 model may be important due to to the potential for individual blood parameters to be affected by comorbidities and medications such as corticosteroids [19, 36, 37].

Several other studies have also described the ability of traditional and/or other modified GAP models to predict outcomes in patients with IPF. For example, incorporating exercise capacity predictors (6MWD and exertional hypoxia) into the traditional GAP model has been shown to improve the predictive value for all-cause mortality (C-statistic 0.756 *versus* 0.683; p=0.014) [15], as has the addition of history of respiratory hospitalisation and 24-week change in FVC in a longitudinal GAP model (C-statistic 0.785 *versus* 0.757) [11]. In another study, incorporating thin-section HRCT-derived semiquantitative fibrotic score into the traditional GAP model significantly improved the predictive value for transplant-free mortality. Among patients with a GAP score ≤3, those with a high fibrotic score had a 4-fold increase in the risk of death or transplantation *versus* those with a low fibrotic score (HR 4.07, p<0.001) [17]. A separate study found that a high serum level of cold-inducible RNA-binding protein predicted greater 1-year IPF progression and all-cause mortality, and that combining the cold-inducible RNA-binding protein and traditional GAP models improved C-statistics compared with either model alone [16]. A modified GAP model developed for survival prediction in East Asian IPF populations by weighting the GAP variables also showed improved performance compared with the traditional GAP model [38]. Lastly, given that the *D*_LCO_ test is not routinely measured in patients with advanced non-small cell lung cancer, a modified GAP model for IPF in non-small cell lung cancer was developed for predicting IPF acute exacerbations and survival based on gender, age, FVC and cancer stage [39]. The current study adds to the existing body of data suggesting that incorporation of additional (molecular and physiological) variables can potentially improve the prognostic value of the traditional GAP model. Further, unlike some of the measures included in previous modified GAP models, blood counts are simple to perform in most patients, meaning that cGAP parameters can be easily obtained and applied for prognostication. WBC and RBC parameters represent clinician-friendly biomarkers that are highly reproducible in everyday clinical practice, considering the availability of largely universal practices for sample collection, processing and interpretation of general blood tests results.

Limitations of this analysis include its *post hoc* nature, meaning that the findings should be considered exploratory in nature. Moreover, patients with severe IPF were excluded; therefore, it is not clear how the predictive value of the cGAP models would compare in patients with more advanced disease. Study outcomes were based on 1-year follow-up, and analyses incorporated baseline variables only. Accordingly, it is not possible to draw conclusions regarding longer-term outcomes or longitudinal changes in WBC or RBC variables. In addition, the influence of steroids on IPF outcomes could not be determined from the datasets used for this analysis because eligibility criteria regarding steroid use varied among the trials; corticosteroid use was prohibited at entry in both CAPACITY and ASCEND as treatment of IPF, but not for other indications unrelated to IPF, and allowed for IPF exacerbations/disease progression during the trials. Finally, other potentially useful predictors, such as exercise capacity parameters and HRCT findings, were not included in the cGAP models.

In conclusion, these findings suggest that the modified cGAP models may be useful tools to help improve prediction of clinical outcomes in patients with IPF. In the future, there may be potential to explore whether a simple and clinically applicable tool could be derived from these models. Additional directions for further research may include development of modified cGAP models for longitudinal use and/or for prediction of treatment response (theragnostic biomarkers).

## Supplementary material

10.1183/23120541.00666-2023.Supp1 **Please note:** supplementary material is not edited by the Editorial Office, and is uploaded as it has been supplied by the author.
Supplementary material 00666-2023.SUPPLEMENT
