# Supplementary material for: Modified blood cell GAP model as a prognostic biomarker in idiopathic pulmonary fibrosis
Source: ERJ Open Res. 2024 Jul 29;10(4):00666-2023. doi: 10.1183/23120541.00666-2023 (PMC11284599; doi:10.1183/23120541.00666-2023)

Modified blood cell GAP model as a prognostic biomarker in idiopathic pulmonary fibrosis

Michael Kreuter,[1] Joyce S Lee,[2] Argyrios Tzouvelekis,[3] Justin M Oldham,[4] Philip L Molyneaux,[5,6] Derek Weycker,[7] Mark Atwood,[7] Katerina Samara,[8] Klaus-Uwe Kirchgässler,[8] Toby M Maher[6,9]

Additional file 1: Supplemental tables and figures

Table S1. Bivariate analyses of white blood cell and red blood cell parameters (categorical)

	n (%)	Mean (SD)	Number of events (% with event) and hazard ratios (95% CI), by study outcome[a]							
			IPF progression		All-cause mortality		All-cause hospitalisation		Respiratory hospitalisation	
**Monocytes, GI/L**										
Low	972 (77.9)	0.4 (0.1)	323 (33.2%)	…	39 (4.0%)	…	153 (15.7%)	…	110 (11.3%)	…
Medium (0.6–<0.95)	253 (20.3)	0.7 (0.1)	98 (38.7%)	1.20 (0.95, 1.50)	23 (9.1%)	2.31 (1.38, 3.88)**	60 (23.7%)	1.57 (1.16, 2.11)**	41 (16.2%)	1.62 (1.13, 2.32)**
High	22 (1.8)	1.1 (0.2)	8 (36.4%)	1.17 (0.58, 2.35)	2 (9.1%)	2.28 (0.55, 9.45)	6 (27.3%)	1.71 (0.76, 3.86)	4 (18.2%)	1.67 (0.62, 4.54)
**Lymphocytes, GI/L**										
Below normal	28 (2.2)	0.8 (0.1)	9 (32.1%)	1.01 (0.52, 1.96)	2 (7.1%)	1.47 (0.36, 6.02)	7 (25.0%)	1.60 (0.75, 3.40)	3 (10.7%)	1.03 (0.33, 3.23)
Normal (1.00–4.80)	1213 (97.3)	2.1 (0.7)	418 (34.5%)	…	62 (5.1%)	…	210 (17.3%)	…	150 (12.4%)	…
Above normal	6 (0.5)	5.2 (0.4)	2 (33.3%)	0.97 (0.24, 3.90)	0 (0.0%)	0.00 (0.00, >999)	2 (33.3%)	1.90 (0.47, 7.65)	2 (33.3%)	2.39 (0.59, 9.65)
**Neutrophils, GI/L**										
Below normal	3 (0.2)	1.1 (0.3)	3 (100.0%)	4.39 (1.41, 13.69)*	0 (0.0%)	0.00 (0.00, >999)	0 (0.0%)	0.00 (0.00, >999)	0 (0.0%)	0.00 (0.00, >999)
Normal (1.70–7.00)	1083 (86.8)	4.7 (1.1)	354 (32.7%)	…	48 (4.4%)	…	175 (16.2%)	…	125 (11.5%)	…
Above normal	161 (12.9)	8.3 (1.7)	72 (44.7%)	1.43 (1.11, 1.85)**	16 (9.9%)	2.31 (1.31, 4.06)**	44 (27.3%)	1.81 (1.30, 2.52)***	30 (18.6%)	1.71 (1.15, 2.55)**
**Basophils, GI/L**										
Below normal	31 (2.5)	0.00 (0.00)	12 (38.7%)	1.15 (0.65, 2.03)	2 (6.5%)	1.26 (0.31, 5.16)	7 (22.6%)	1.36 (0.64, 2.88)	4 (12.9%)	1.08 (0.40, 2.93)
Normal (0.01–0.30)	1216 (97.5)	0.06 (0.03)	417 (34.3%)	…	62 (5.1%)	…	212 (17.4%)	…	151 (12.4%)	…
Above normal	0 (0.0)	…	…	…	…	…	…	…	…	…
**Eosinophils, GI/L**										
Below normal	23 (1.8)	0.0 (0.0)	8 (34.8%)	1.02 (0.51, 2.06)	1 (4.3%)	0.85 (0.12, 6.17)	4 (17.4%)	0.94 (0.35, 2.54)	2 (8.7%)	0.61 (0.15, 2.46)
Normal (0.05–0.50)	1141 (91.5)	0.2 (0.1)	383 (33.6%)	…	56 (4.9%)	…	201 (17.6%)	…	142 (12.4%)	…
Above normal	83 (6.7)	0.7 (0.2)	38 (45.8%)	1.55 (1.11, 2.17)*	7 (8.4%)	1.74 (0.79, 3.82)	14 (16.9%)	0.98 (0.57, 1.69)	11 (13.3%)	1.20 (0.65, 2.22)
**Mean corpuscular Hb,**										
**pg**	33 (2.6)	24.9 (1.5)	10 (30.3%)	0.84 (0.45, 1.58)	3 (9.1%)	1.93 (0.60, 6.26)	11 (33.3%)	2.14 (1.16, 3.96)*	9 (27.3%)	2.20 (1.11, 4.35)*
Below normal	802 (64.3)	29.8 (1.1)	284 (35.4%)	…	38 (4.7%)	…	148 (18.5%)	…	105 (13.1%)	…
Normal (27.0–31.0)	412 (33.0)	32.7 (1.1)	135 (32.8%)	0.91 (0.74, 1.12)	23 (5.6%)	1.19 (0.71, 1.99)	60 (14.6%)	0.77 (0.57, 1.04)	41 (10.0%)	0.80 (0.56, 1.14)
Above normal										

**Mean corpuscular Hb concentration, g/L**										
Below normal	86 (6.9)	309.7 (4.2)	32 (37.2%)	1.11 (0.78, 1.60)	6 (7.0%)	1.42 (0.61, 3.30)	21 (24.4%)	1.51 (0.96, 2.37)	17 (19.8%)	1.56 (0.94, 2.58)
Normal (320.0–360.0)	1153 (92.5)	336.3 (11.1)	393 (34.1%)	…	56 (4.9%)	…	195 (16.9%)	…	137 (11.9%)	…
Above normal	8 (0.6)	370.0 (0.0)	4 (50.0%)	1.71 (0.64, 4.58)	2 (25.0%)	6.06 (1.48, 24.84)*	3 (37.5%)	2.63 (0.84, 8.24)	1 (12.5%)	1.92 (0.27, 13.95)
**Erythrocytes, TI/L**										
Below normal	411 (33.0)	4.1 (0.4)	146 (35.5%)	1.08 (0.88, 1.32)	28 (6.8%)	1.64 (0.99, 2.70)	86 (20.9%)	1.36 (1.03, 1.79)*	58 (14.1%)	1.26 (0.91, 1.75)
Normal (4.3–6.0)	807 (64.7)	4.9 (0.3)	276 (34.2%)	…	34 (4.2%)	…	127 (15.7%)	…	93 (11.5%)	…
Above normal	29 (2.3)	5.8 (0.3)	7 (24.1%)	0.68 (0.32, 1.43)	2 (6.9%)	1.68 (0.40, 7.01)	6 (20.7%)	1.34 (0.59, 3.04)	4 (13.8%)	1.25 (0.46, 3.41)
**Hb (g/L)**										
Below normal	453 (36.3)	127.7 (8.2)	166 (36.6%)	1.17 (0.94, 1.45)	30 (6.6%)	1.56 (0.90, 2.70)	96 (21.2%)	1.48 (1.10, 2.01)*	69 (15.2%)	1.49 (1.04, 2.13)*
Normal (121.0–172.0)	510 (40.9)	144.3 (4.0)	168 (32.9%)	…	22 (4.3%)	…	76 (14.9%)	…	52 (10.2%)	…
Above normal	284 (22.8)	158.7 (6.1)	95 (33.5%)	0.98 (0.77, 1.27)	12 (4.2%)	0.97 (0.48, 1.96)	47 (16.5%)	1.11 (0.77, 1.60)	34 (12.0%)	1.23 (0.80, 1.90)
**Platelets, GI/L**										
Below normal	40 (3.2)	127.6 (19.6)	12 (30.0%)	0.86 (0.49, 1.53)	1 (2.5%)	0.47 (0.06, 3.36)	5 (12.5%)	0.68 (0.28, 1.65)	5 (12.5%)	0.98 (0.40, 2.39)
Normal (150.0–450.0)	1202 (96.4)	249.5 (58.3)	415 (34.5%)	…	63 (5.2%)	…	212 (17.6%)	…	149 (12.4%)	…
Above normal	5 (0.4)	524.8 (72.0)	2 (40.0%)	1.16 (0.29, 4.67)	0 (0.0%)	0.00 (0.00, >999)	2 (40.0%)	2.47 (0.61, 9.96)	1 (20.0%)	2.25 (0.31, 16.12)

[a]Follow-up between visits was 12–13 weeks

**P*<0.05; ***P*<0.01; ****P*<0.001

GI, 10^9; Hb, haemoglobin; IPF, idiopathic pulmonary fibrosis; SD, standard deviation; TI, 10^{12}

Table S2. Multivariable analyses of white blood cell/red blood cell parameters (continuous) and GAP parameters

	Hazard ratios (95% CI), by study outcome			
Model specification	IPF progression	All-cause mortality	All-cause hospitalisation	Respiratory hospitalisation
**GAP**				
Age	1.00 (0.99, 1.01)	1.02 (0.99, 1.06)	1.01 (0.99, 1.03)	1.00 (0.99, 1.03)
Gender (male vs female)	0.92 (0.74, 1.15)	1.67 (0.85, 3.29)	0.68 (0.51, 0.90)**	0.76 (0.54, 1.07)
FVC, % predicted	0.99 (0.98, 1.00)	0.98 (0.96, 1.01)	0.99 (0.98, 1.01)	0.99 (0.98, 1.01)
DLco, % predicted	0.97 (0.96, 0.99)***	0.92 (0.88, 0.95)***	0.96 (0.94, 0.98)***	0.95 (0.93, 0.97)***
**GAP + monocyte counts (cGAP1)**				
Age	1.00 (0.99, 1.01)	1.02 (0.98, 1.05)	1.01 (0.99, 1.03)	1.01 (0.98, 1.03)
Gender (male vs female)	0.92 (0.74, 1.15)	1.58 (0.80, 3.11)	0.64 (0.48, 0.86)**	0.73 (0.52, 1.03)
FVC, % predicted	0.99 (0.98, 1.00)	0.99 (0.96, 1.01)	0.99 (0.98, 1.01)	1.00 (0.98, 1.01)
DLco, % predicted	0.97 (0.96, 0.99)***	0.92 (0.88, 0.95)***	0.96 (0.94, 0.98)***	0.95 (0.93, 0.97)***
Monocytes	1.04 (0.58, 1.85)	5.96 (1.77, 20.07)**	2.90 (1.42, 5.89)**	3.16 (1.36, 7.36)**
**GAP + monocyte counts + other WBC and RBC parameters (cGAP2)**				
Age				
Gender (male vs female)	1.00 (0.98, 1.01)	1.01 (0.98, 1.05)	1.01 (0.99, 1.03)	1.00 (0.98, 1.02)
FVC, % predicted	0.98 (0.76, 1.26)	2.00 (0.94, 4.25)	0.76 (0.54, 1.06)	0.85 (0.57, 1.28)
DLco, % predicted	0.99 (0.98, 1.00)	0.99 (0.96, 1.01)	1.00 (0.98, 1.01)	1.00 (0.98, 1.01)
Monocytes	0.98 (0.96, 0.99)***	0.92 (0.89, 0.96)***	0.96 (0.94, 0.98)***	0.95 (0.93, 0.97)***
Lymphocytes	0.71 (0.37, 1.37)	3.83 (0.91, 16.18)	2.27 (0.99, 5.20)	2.63 (0.98, 7.05)
Neutrophils	0.91 (0.79, 1.06)	0.83 (0.56, 1.23)	0.99 (0.81, 1.21)	1.01 (0.80, 1.28)
Basophils	1.07 (1.01, 1.13)*	1.11 (0.98, 1.26)	1.06 (0.98, 1.15)	1.08 (0.98, 1.19)
Eosinophils	6.12 (0.26, 146.54)	0.52 (0.00, 2829.26)	1.47 (0.01, 158.25)	0.50 (0.00, 178.70)
Mean corpuscular Hb	1.88 (1.07, 3.31)*	1.56 (0.39, 6.31)	1.00 (0.43, 2.33)	1.24 (0.44, 3.45)

Mean corpuscular Hb concentration	1.02 (0.96, 1.08)	1.04 (0.89, 1.20)	0.95 (0.88, 1.03)	0.94 (0.85, 1.03)
Erythrocytes	0.99 (0.98, 1.00)	1.00 (0.98, 1.02)	1.00 (0.99, 1.01)	1.00 (0.99, 1.02)
Hb	0.90 (0.73, 1.10)	0.99 (0.67, 1.47)	0.98 (0.78, 1.23)	0.99 (0.79, 1.24)
Platelets	1.00 (0.99, 1.01)	0.98 (0.95, 1.00)	0.99 (0.98, 1.01)	0.99 (0.97, 1.00)
	1.00 (1.00, 1.00)	1.00 (1.00, 1.00)	1.00 (1.00, 1.00)	1.00 (1.00, 1.00)

*$P<0.05$; **$P<0.01$; ***$P<0.001$

cGAP, modified GAP; CI, confidence interval; DLco, carbon monoxide diffusing capacity; FVC, forced vital capacity; GAP, Gender, Age and Physiology; Hb, haemoglobin; IPF, idiopathic pulmonary fibrosis; RBC, red blood cell; WBC, white blood cell

Table S3. Multivariable analyses of white blood cell/red blood cell parameters (categorical) and GAP parameters

Model Specification	Number of events (% with event) and hazard ratios (95% CI), by study outcome							
	IPF progression[a]		All-cause mortality[b]		All-cause hospitalisation[c]		Respiratory hospitalisation[d]	
**GAP**								
Age	427	1.00 (0.99, 1.01)	64	1.02 (0.99, 1.06)	219	1.01 (0.99, 1.03)	155	1.01 (0.99, 1.03)
Gender (male vs female)	427	0.92 (0.74, 1.15)	64	1.67 (0.85, 3.29)	219	0.68 (0.51, 0.90)**	155	0.76 (0.54, 1.07)
FVC, % predicted	427	0.99 (0.98, 1.00)	64	0.98 (0.96, 1.01)	219	0.99 (0.98, 1.01)	155	0.99 (0.98, 1.01)
DLco, % predicted	427	0.97 (0.96, 0.98)***	64	0.92 (0.88, 0.95)***	219	0.96 (0.94, 0.98)***	155	0.95 (0.93, 0.97)***
**GAP + monocyte counts (cGAP1)[a]**								
Age	427	1.00 (0.99, 1.01)	64	1.02 (0.98, 1.05)	219	1.01 (0.99, 1.03)	155	1.01 (0.98, 1.03)
Gender (male vs female)	427	0.92 (0.74, 1.14)	64	1.65 (0.84, 3.26)	219	0.66 (0.50, 0.88)**	155	0.75 (0.53, 1.06)
FVC, % predicted	427	0.99 (0.98, 1.00)	64	0.99 (0.96, 1.01)	219	0.99 (0.98, 1.01)	155	0.99 (0.98, 1.01)
DLco, % predicted	427	0.97 (0.96, 0.99)***	64	0.92 (0.88, 0.95)***	219	0.96 (0.94, 0.98)***	155	0.95 (0.93, 0.97)***
Monocytes								
0.60 –<0.95 GI/L	97	1.11 (0.88, 1.40)	23	1.87 (1.11, 3.16)*	60	1.44 (1.07, 1.95)*	41	1.47 (1.03, 2.12)*
≥0.95 GI/L	8	1.11 (0.55, 2.25)	2	2.01 (0.48, 8.38)	6	1.76 (0.78, 4.00)	4	1.73 (0.63, 4.71)
**GAP + monocyte counts + other WBC and RBC parameters (cGAP2)[a]**								
Age	427	1.00 (0.98, 1.01)	64	1.01 (0.97, 1.04)	219	1.01 (0.99, 1.03)	155	1.01 (0.99, 1.03)
Gender (male vs female)	427	0.99 (0.78, 1.26)	64	2.28 (1.11, 4.70)*	219	0.77 (0.56, 1.07)	155	0.85 (0.58, 1.26)
FVC, % predicted	427	0.99 (0.98, 1.00)*	64	0.99 (0.97, 1.01)	219	1.00 (0.98, 1.01)	155	1.00 (0.98, 1.01)
DLco, % predicted	427	0.98 (0.96, 0.99)***	64	0.92 (0.88, 0.95)***	219	0.96 (0.94, 0.98)***	155	0.95 (0.93, 0.97)***
Monocytes								
0.60 –<0.95 GI/L	97	1.08 (0.85, 1.37)	23	1.81 (1.04, 3.17)*	60	1.38 (1.01, 1.90)*	41	1.38 (0.94, 2.02)
≥0.95 GI/L	8	0.95 (0.46, 1.96)	2	1.80 (0.40, 8.04)	6	1.67 (0.71, 3.91)	4	1.44 (0.50, 4.09)

Lymphocytes								
Below Normal	9	0.95 (0.48, 1.88)	2	0.95 (0.21, 4.31)	7	1.29 (0.55, 3.01)	3	0.83 (0.24, 2.85)
Above Normal	2	0.92 (0.22, 3.94)	0	0.00 (0.00, >999)	2	1.43 (0.32, 6.34)	2	2.28 (0.51, 10.09)
Neutrophils								
Below Normal	3	4.80 (1.33, 17.28)*	0	0.00 (0.00, >999)	0	0.00 (0.00, >999)	0	0.00 (0.00, >999)
Above Normal	72	1.26 (0.96, 1.64)	16	1.64 (0.90, 3.00)	44	1.40 (0.98, 2.00)	30	1.32 (0.86, 2.03)
Basophils								
Below Normal	12	0.83 (0.44, 1.55)	2	0.61 (0.13, 2.95)	7	0.98 (0.43, 2.24)	4	0.88 (0.31, 2.49)
Above Normal	---	---	---	---	---	---	---	---
Eosinophils								
Below Normal	8	0.96 (0.45, 2.08)	1	0.78 (0.09, 6.58)	4	0.66 (0.21, 2.08)	2	0.51 (0.11, 2.32)
Above Normal	38	1.46 (1.03, 2.05)*	7	1.17 (0.51, 2.69)	14	0.83 (0.48, 1.45)	11	1.04 (0.56, 1.94)
Mean corpuscular Hb								
Below Normal	10	0.79 (0.41, 1.54)	3	2.17 (0.56, 8.36)	11	2.19 (1.09, 4.40)*	9	2.36 (1.10, 5.09)*
Above Normal	134	0.87 (0.68, 1.10)	23	0.94 (0.51, 1.74)	60	0.67 (0.48, 0.95)*	41	0.73 (0.49, 1.10)
Mean corpuscular Hb concentration								
Below Normal	32	1.06 (0.72, 1.56)	6	1.04 (0.41, 2.65)	21	1.09 (0.66, 1.79)	17	1.20 (0.69, 2.08)
Above Normal	4	1.42 (0.52, 3.87)	2	3.49 (0.79, 15.33)	3	2.81 (0.88, 9.02)	1	1.85 (0.25, 13.60)
Erythrocytes								
Below Normal	146	1.04 (0.78, 1.37)	28	1.43 (0.70, 2.92)	86	1.43 (0.98, 2.09)	58	1.34 (0.86, 2.11)
Above Normal	7	0.65 (0.30, 1.41)	2	1.55 (0.34, 7.12)	6	1.09 (0.46, 2.59)	4	1.01 (0.36, 2.87)
Hb								
Below Normal	166	1.18 (0.90, 1.54)	30	1.70 (0.85, 3.39)	96	1.09 (0.74, 1.59)	69	1.15 (0.73, 1.80)
Above Normal	95	1.05 (0.80, 1.37)	12	0.97 (0.45, 2.07)	47	1.24 (0.84, 1.83)	34	1.35 (0.85, 2.14)
Platelets								
Below Normal	12	0.89 (0.49, 1.63)	1	0.59 (0.08, 4.34)	5	0.83 (0.34, 2.06)	5	1.28 (0.52, 3.19)
Above Normal	2	0.82 (0.20, 3.41)	0	0.00 (0.00, >999)	2	1.75 (0.41, 7.58)	1	1.45 (0.19, 11.13)

[a]Reference group = Normal, except for monocytes where reference group = <0.60 GI/L

**P*<0.05; ***P*<0.01; ****P*<0.001

cGAP, modified GAP; CI, confidence interval; DLco, carbon monoxide diffusing capacity; FVC, forced vital capacity; GAP, Gender, Age and Physiology; GI, 10^9; Hb, haemoglobin; IPF, idiopathic pulmonary fibrosis; RBC, red blood cell; WBC, white blood cell

Table S4. Multivariable analyses of white blood cell/red blood cell (categorical) and other parameters (full set of results)

	Hazard ratios (95% CI), by study outcome			
Independent variables[a,b]	IPF progression	All-cause mortality	All-cause hospitalisation	Respiratory hospitalisation
**Monocytes**				
0.60–<0.95 GI/L	1.05 (0.82, 1.34)	1.57 (0.89, 2.78)	1.32 (0.95, 1.83)	1.17 (0.79, 1.74)
≥0.95 GI/L	1.02 (0.49, 2.11)	1.85 (0.40, 8.42)	1.13 (0.48, 2.66)	0.92 (0.32, 2.62)
**Lymphocytes**				
Below normal	0.96 (0.49, 1.90)	1.03 (0.22, 4.91)	1.06 (0.43, 2.61)	0.36 (0.08, 1.59)
Above normal	0.88 (0.20, 3.86)	0.00 (0.00, >999)	1.40 (0.32, 6.21)	2.44 (0.54, 11.04)
**Neutrophils**				
Below normal	4.68 (1.29, 16.94)*	0.00 (0.00, >999)	0.00 (0.00, >999)	0.00 (0.00, >999)
Above normal	1.21 (0.92, 1.60)	1.08 (0.57, 2.06)	1.30 (0.91, 1.88)	1.24 (0.80, 1.92)
**Basophils**				
Below normal	0.91 (0.48, 1.75)	0.55 (0.11, 2.74)	1.09 (0.49, 2.45)	0.89 (0.31, 2.52)
Above normal	…	…	…	…
**Eosinophils**				
Below normal	1.07 (0.49, 2.36)	0.58 (0.06, 5.35)	0.73 (0.24, 2.19)	0.65 (0.15, 2.86)
Above normal	1.53 (1.08, 2.17)*	1.37 (0.59, 3.15)	0.86 (0.49, 1.51)	0.99 (0.53, 1.87)
**Mean corpuscular Hb in pg**				
Below normal	0.91 (0.47, 1.77)	3.56 (0.92, 13.78)	2.36 (1.18, 4.72)*	2.80 (1.29, 6.07)**
Above normal	0.91 (0.71, 1.16)	0.94 (0.51, 1.76)	0.63 (0.44, 0.89)**	0.65 (0.42, 0.99)*
**Mean corpuscular Hb concentration in g/L**				

Below normal	0.98 (0.66, 1.47)	0.75 (0.26, 2.10)	0.95 (0.57, 1.58)	1.19 (0.67, 2.09)
Above normal	1.31 (0.48, 3.55)	6.80 (1.52, 30.42)*	2.13 (0.67, 6.85)	0.89 (0.12, 6.55)
**Erythrocytes**				
Below normal	1.01 (0.76, 1.34)	1.48 (0.69, 3.15)	1.45 (0.99, 2.14)	1.27 (0.79, 2.02)
Above normal	0.73 (0.33, 1.58)	1.47 (0.31, 6.92)	1.19 (0.50, 2.83)	1.08 (0.38, 3.09)
**Hb**				
Below normal	1.11 (0.85, 1.46)	1.28 (0.62, 2.64)	1.08 (0.73, 1.60)	1.16 (0.72, 1.85)
Above normal	0.93 (0.71, 1.23)	0.87 (0.40, 1.89)	1.18 (0.80, 1.74)	1.19 (0.75, 1.89)
**Platelets**				
Below normal	0.79 (0.42, 1.48)	0.42 (0.06, 3.14)	0.70 (0.28, 1.73)	1.09 (0.43, 2.72)
Above normal	0.89 (0.21, 3.77)	0.00 (0.00, >999)	1.80 (0.42, 7.77)	1.13 (0.14, 8.89)
**Baseline %FVC**	0.99 (0.98, 1.00)*	…	…	…
**Baseline %DLco**	0.98 (0.97, 0.99)**	0.94 (0.90, 0.97)***	0.97 (0.95, 0.98)***	0.97 (0.95, 0.99)**
**Pirfenidone**	0.61 (0.50, 0.74)***	0.47 (0.27, 0.81)**	…	…
**Pulmonary hypertension**	1.82 (1.10, 3.00)*	…	1.86 (1.03, 3.33)*	2.04 (1.05, 3.98)*
**Gastroesophageal reflux disease**	0.80 (0.66, 0.98)*	…	…	…
**Congestive heart failure**	2.22 (1.03, 4.79)*	…	3.57 (1.69, 7.56)***	4.64 (1.92, 11.81)***
**Steroid use**	1.32 (1.06, 1.64)*	3.91 (1.82, 8.38)***	1.97 (1.42, 2.73)***	3.39 (2.15, 5.32)***
**Baseline 6MWD**	…	1.00 (0.99, 1.00)**	…	…
**Male**	…	2.98 (1.38, 6.43)**	…	…

**Smoker (current/former)**	…	2.36 (1.21, 4.61)*	…	…
**Obesity**[c]	…	0.51 (0.29, 0.89)*	…	…
**UCSD-SOBQ**	…	…	1.01 (1.00, 1.02)**	1.01 (1.00, 1.02)*
**Coronary artery disease**	…	…	1.56 (1.13, 2.14)**	1.88 (1.29, 2.73)**
**Hypercholesterolemia**	…	…	0.70 (0.52, 0.94)*	0.58 (0.41, 0.83)**

[a]Blood cell variables were not forced into the model; backwards selection was employed to identify all independent variables (*P*<0.10). Follow-up between visits was 12–13 weeks; model specifications determined via backward selection from the following candidate variables: treatment (pirfenidone vs placebo), age, male, current/former smoker, FVC (% predicted), DLco, 6MWD, UCSD-SOBQ, pulmonary hypertension, pulmonary embolism, gastroesophageal reflux disease; deep vein thrombosis, chronic obstructive pulmonary disease, arteriosclerosis, chronic renal failure, coronary artery disease, myocardial infarction, chronic heart failure, hypertension, hypercholesterolaemia, obesity, infections, steroid use

[b]Reference group = Normal, except for monocytes where reference group = <0.60 GI/L

[c]Defined as BMI >30kg/m^2

**P*<0.05; ***P*<0.01; ****P*<0.001

6MWD, 6-minute walk distance; BMI, body mass index; CI, confidence interval; DLco, carbon monoxide diffusing capacity; FVC, forced vital capacity; GI, 10^9; Hb, haemoglobin; IPF, idiopathic pulmonary fibrosis; UCSD-SOBQ, University of California—San Diego Shortness of Breath Questionnaire

Table S5. Multivariable analyses of white blood cell/red blood cell (continuous) and other parameters (full set of results)

	Hazard ratios (95% CI), by study outcome			
Independent variables[a]	IPF progression[b]	All-cause mortality[c]	All-cause hospitalisation[d]	Respiratory hospitalisation[e]
**Monocytes, GI/L**	0.77 (0.40, 1.50)	4.14 (0.85, 20.19)	1.63 (0.70, 3.79)	1.53 (0.56, 4.18)
**Lymphocytes, GI/L**	0.91 (0.79, 1.06)	0.83 (0.56, 1.24)	1.01 (0.83, 1.23)	1.10 (0.88, 1.39)
**Neutrophils, GI/L**	1.06 (1.00, 1.13)*	1.06 (0.92, 1.22)	1.06 (0.97, 1.15)	1.07 (0.97, 1.18)
**Basophils, GI/L**	5.93 (0.22, 158.05)	0.94 (0.00, >999)	2.80 (0.02, 327.96)	2.98 (0.01, 922.07)
**Eosinophils, GI/L**	2.14 (1.20, 3.80)**	2.08 (0.52, 8.30)	0.94 (0.39, 2.28)	0.81 (0.27, 2.47)
**Mean corpuscular Hb, pg**	1.04 (0.98, 1.10)	1.01 (0.86, 1.19)	0.93 (0.85, 1.01)	0.89 (0.80, 0.99)*
**Mean corpuscular Hb, g/L**	1.00 (0.99, 1.00)	1.01 (0.99, 1.03)	1.00 (0.99, 1.01)	0.99 (0.98, 1.01)
**Erythrocytes, TI/L**	1.01 (0.84, 1.22)	1.01 (0.61, 1.69)	0.93 (0.69, 1.26)	0.89 (0.60, 1.34)
**Hb, g/L**	0.99 (0.98, 1.00)	0.98 (0.95, 1.01)	0.99 (0.98, 1.01)	0.99 (0.97, 1.01)
**Platelets, GI/L**	1.00 (1.00, 1.00)	1.00 (1.00, 1.01)	1.00 (1.00, 1.00)	1.00 (1.00, 1.00)
**Baseline %FVC**	0.99 (0.98, 1.00)*	…	…	…
**Baseline %DLco**	0.98 (0.97, 0.99)***	0.94 (0.91, 0.98)**	0.97 (0.95, 0.98)***	0.97 (0.95, 0.99)**
**Pirfenidone**	0.61 (0.50, 0.74)***	0.48 (0.28, 0.82)**	…	…
**Pulmonary hypertension**	1.92 (1.17, 3.16)*	…	2.00 (1.12, 3.55)*	2.01 (1.04, 3.89)*

**Gastroesophageal reflux disease**	0.81 (0.66, 0.99)*	…	…	…
**Congestive heart failure**	2.29 (1.07, 4.94)*	4.40 (1.01, 19.15)*	3.43 (1.64, 7.16)**	3.86 (1.64, 9.06)**
**Steroid use**	1.35 (1.09, 1.67)**	3.95 (1.84, 8.48)***	1.98 (1.43, 2.73)***	3.32 (2.12, 5.18)***
**Baseline 6MWD**	…	1.00 (0.99, 1.00)**	…	…
**Male**	…	2.76 (1.23, 6.19)*	…	…
**Smoker (current/former)**	…	2.28 (1.18, 4.44)*	…	…
**Obesity**[c]	…	0.50 (0.28, 0.89)*	…	…
**UCSD-SOBQ**	…	…	1.01 (1.00, 1.02)**	1.01 (1.00, 1.02)*
**Coronary artery disease**	…	…	1.53 (1.11, 2.10)**	1.87 (1.28, 2.72)**
**Hypercholesterolemia**	…	…	0.72 (0.54, 0.97)*	0.58 (0.41, 0.83)**

N=1247 for all variables. Follow-up between visits was 12–13 weeks

[a]All blood cell variables were forced into the model. Other model specifications were determined via backward selection from the following candidate variables: treatment (pirfenidone vs placebo), male, current/former smoker, FVC (% predicted, time-dependent), DLco (% predicted, time-dependent), 6MWD (time-dependent), UCSD-SOBQ (time-dependent), pulmonary hypertension, pulmonary embolism, gastroesophageal reflux disease, deep vein thrombosis, chronic obstructive pulmonary disease, arteriosclerosis, chronic renal failure, coronary artery disease, myocardial infarction, chronic heart failure, hypertension, hypercholesterolaemia, obesity, infections, steroid use

[b]Number of events (% with the event) = 429 (34.4%) for IPF progression

[c]Number of events (% with the event) = 64 (5.1%) for all-cause mortality

[d]Number of events (% with the event) = 219 (17.6%) for all-cause hospitalisation

[e]Number of events (% with the event) = 155 (12.4%) for respiratory hospitalisation

**P*<0.05; ***P*<0.01; ****P*<0.001

6MWD, 6-minute walk distance; CI, confidence interval; DLco, carbon monoxide diffusing capacity; FVC, forced vital capacity; GI, 10^9; Hb, haemoglobin; IPF, idiopathic pulmonary fibrosis; TI, 10^{12}; UCSD-SOBQ, University of California—San Diego Shortness of Breath Questionnaire

Figure S1. Kaplan–Meier curves for all-cause mortality, by tertile scores for the (a) Gender, Age, Physiology, (b) modified Gender, Age, Physiology 1 and (c) modified Gender, Age, Physiology 2 models

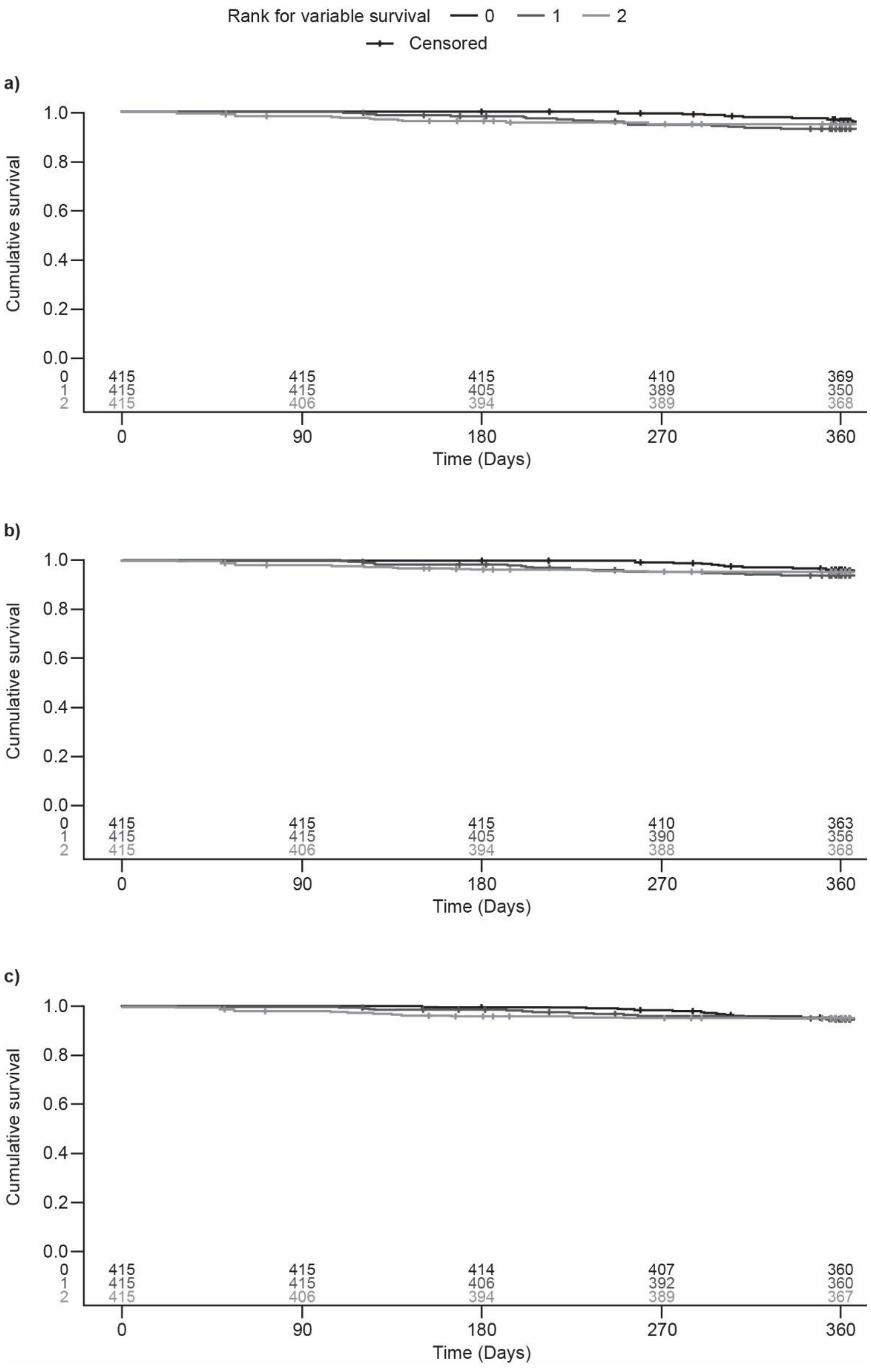

Supplement: Supplementary file 1 [file 00666-2023.SUPPLEMENT.pdf]
